# Managing the Demand for Global Health Education

**DOI:** 10.1371/journal.pmed.1001118

**Published:** 2011-11-08

**Authors:** Vanessa B. Kerry, Thumbi Ndung'u, Rochelle P. Walensky, Patrick T. Lee, V. Frederick I. B. Kayanja, David R. Bangsberg

**Affiliations:** 1 Center for Global Health, Massachusetts General Hospital, Boston, Massachusetts, United States of America; 2 Harvard Medical School, Boston, Massachusetts, United States of America; 3 Division of Pulmonary and Critical Care, Department of Medicine, Massachusetts General Hospital, Boston, Massachusetts, United States of America; 4 HIV Pathogenesis Programme, Doris Duke Medical Research Institute, Nelson R Mandela School of Medicine, University of KwaZulu-Natal, Durban, South Africa; 5 Division of General Medicine, Department of Medicine, Massachusetts General Hospital, Boston, Massachusetts, United States of America; 6 Division of Infectious Disease, Department of Medicine, Massachusetts General Hospital, Boston, Massachusetts, United States of America; 7 Division of Infectious Disease, Department of Medicine, Brigham and Women's Hospital, Boston, Massachusetts, United States of America; 8 Mbarara University of Science and Technology, Mbarara, Uganda

## Abstract

Vanessa Kerry and colleagues discuss how to manage the unprecedented growth in and demand for global health programs in the United States, Europe and other high-income countries.

**Summary Points**

- Emerging training programs in global health worldwide create a unique opportunity to re-examine the strategy to scale-up human resources to reduce the global burden of disease.
- Funding should be channeled to programs that promise sustained, rational, and effective training and that cultivate the best available talent in all settings.
- Students and trainees require evidence of and mentorship toward reproducible and successful career pathways in all clinical, research, and training aspects of global health.
- Global health training programs should be evaluated by the quality of the experience for trainees from all settings and by the incremental improvement in in-country care, infrastructure, and/or research.

Globalization has opened access to distant regions of the world and increased awareness of global health disparities [1]–[3]. This heightened awareness, coupled with the rapid expansion of treatment to over 5 million people with HIV worldwide [4], has captured the imagination of a growing generation of health professionals who are motivated to make a difference across international boundaries. Their enthusiasm has fueled an unprecedented growth in academic global health programs in higher-income (HI) countries partnering with programs in low- and middle-income countries (LMICs) countries [5]–[7]. How do we manage this explosive growth to most effectively and sustainably reduce the global burden of disease?

The exponentially growing investment in global health training is an opportunity to reexamine our strategy and goals. Investments should expand beyond the needs of the universities in HI countries, which already dominate global health leadership, towards mutually beneficial partnerships that leverage the best available talent across the globe. This approach will require re-prioritizing existing resources and identifying new funding opportunities to build public health and health science leadership. Balanced partnerships, based on joint goals and measures, provide an outlet for growing enthusiasm in HI countries while also creating opportunities for health system strengthening, innovation, and leadership development in LMICs.

## Managing the Surge in Interest

Global health derives its roots from tropical medicine, which was founded as a field in 19th century colonial Europe [8]. More recently, the science and delivery of global health has evolved into a well-defined discipline [9],[10]. Koplan et al. distinguish global health as: 1) referring to any health issue that concerns many countries or is affected by transnational determinants; 2) referring to a scope of problems versus geography; 3) encompassing the complex interactions between societies; 4) using the resources, knowledge, and experience of diverse societies to address health challenges around the globe and; 5) embracing prevention, treatment, rehabilitation, and “other aspects of clinical medicine” and basic science [7]. Notably, this definition includes social, economic, environmental, and political determinants of population health, and a science to optimize individual patient care.

In higher education institutions around the world, the demand for global health training opportunities abroad in myriad clinical disciplines is soaring [11]–[14]. This interest occurs at all levels [14]–[19]. The International Federation of Medical Students, representing 1.2 million medical students from 91 countries, openly calls for medical schools to ensure a comprehensive global health framework within their curriculum [15]. At the graduate medical level, growth in interest has been documented in surgery, internal medicine, pediatrics, and family medicine residencies [14],[20]–[22]. Universities, and their medical education and training programs, are hurrying to keep pace with the demand. A recent survey by the Consortium for Universities for Global Health found that the number of university-based global health programs in North America has more than quadrupled from eight to over 40 between 2003 and 2009 [6].

The number and breadth of programs is well documented among medical schools from North America, Europe, South America, and the Pacific [11],[15]–[19],[23]. At the graduate medical education level, the number of programs is also growing, though the literature is most robust for North American institutions. Sixty-one graduate medical education programs in the United States in a national survey offered international electives and 11 programs had specified global health tracks as of 2005 [24]. This growth is fueled by the moral imperative to improve public health worldwide, as well as by a competitive effort to attract the top applicants. Recent surveys of aspiring residents in emergency medicine and family medicine indicated that students who had participated in global health activities during medical school ranked graduate medical programs with global health rotations over those without such offerings [25],[26].

Existing programs reflect a diversity of mission and education experience, which manifest in alternative structures, areas of focus, partnerships, and degree of knowledge exchange with resource-limited settings. The full spectrum of geography, clinical specialty, program size and content, or character of exchange and partnership remain undocumented. Organizations such as the Global Health Education Consortium and the Association of Schools of Public Health in the European Region, for example, have developed core competencies for global health education for both undergraduate and graduate medical education programs to address this heterogeneity across programs [27]–[31]. Similar efforts are in process for non-clinical training programs, including research-, service-, and programmatic-based global health education [30],[32]. These programs must not only choose to endorse these standards but also to then define a mutually acceptable accreditation process.

## Balancing Enthusiasm in HI Countries with Retention in LMIC Settings

**Figure 1:**
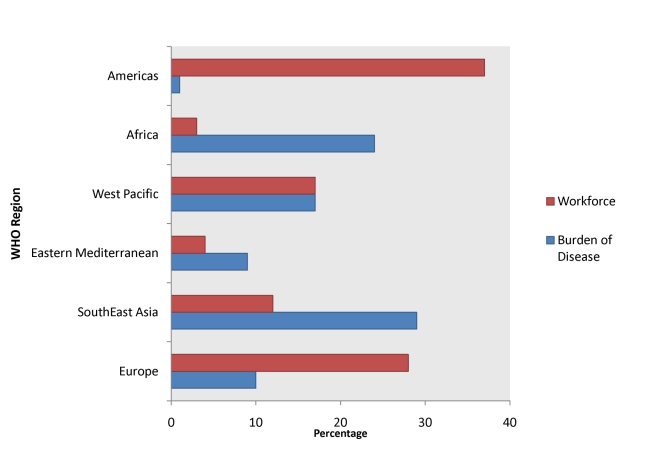
Global burden of disease versus workforce [33],[34].

The growing number of medical trainees in HI countries seeking “in-country” training experiences in LMIC settings is ironically counterbalanced by a growing number of trainees who leave LMIC areas for more infrastructure-replete practice settings. According to the World Health Organization (WHO), 57 countries need more than 2.4 million additional doctors, nurses, and midwives [33]. Unfortunately, the regions with most severe health care shortages are the same regions with the highest burden of disease. For example, Africa has 24% of the global burden of disease, but only 3% of the global health care workforce, and only 1% of the world's health expenditure (Figure 1) [33],[34]. This shortage is driven, first, by insufficient training capacity and, second, by higher salaries, better working conditions, and more advanced training opportunities in HI settings.

Doctors from HI countries have a growing enthusiasm to work in LMIC settings; however, sending health professionals from wealthier settings is not a sustainable or efficient approach to fill professional gaps in developing countries. Health professionals from wealthier countries often require sufficient salaries to cover educational debt or other costs [35]. Limited public health funds that could be directed to medical treatment, in-country workforce expansion, and/or infrastructure development are used sub-optimally. The agenda for many of these health professionals from wealthier countries is often shorter-term than their in-country counterparts. Professionals from wealthier nations generally have increased opportunities for mobility and career development, or are lured by prior roots in their country of origin. No matter how well-meaning or energetic, brief tenure does not create a nuanced understanding of a disease in a developing setting.

**Figure 2:**
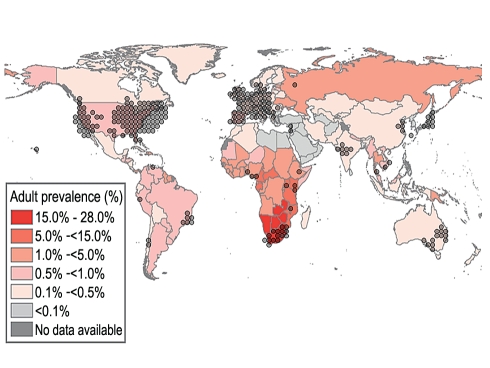
Global HIV prevalence [36] overlaid with 300 PubMed articles (keyword: HIV) published in 2007 on HIV. The first (chronologically) 300 articles published in 2007 on HIV listed by PubMed (keyword: HIV) were mapped by the home institution of the first author (or corresponding author). Of the 300 mapped publications, 37% came from North America while 21% came from Western Europe. Sub-Saharan Africa, in contrast, represents just 8% of the publications.

Career development is paramount. Currently, there is insufficient senior leadership in developing countries to help guide research, address local resource constraints, or mentor all interested trainees from *either* side of a partnership. For example, while there are many leaders in LMIC settings who have expertly championed international initiatives, the highest concentration of global health leadership measured by academic publications resides in HI regions with the lowest burden of disease (Figure 2) [36]. While many factors, including job security, safety, or wages, influence migration of indigenous health care professionals from LMIC health systems, lack of career mobility or training opportunities also influences emigration [33],[37],[38]. In Cameroon, lack of opportunities or promotion, and desire to gain advanced training, ranked above poor wages as reasons why health care professionals chose to migrate [37]. Health professionals who leave for training but return may have needed skills but do not have the needed infrastructure and support to practice their trade nor facile access to international academic discourse [39],[40]. More constructive investments in research and training in resource-limited settings, such as those spearheaded by the Wellcome Trust, the International Association of Public Health Institutes, or the Third World Academy of Sciences, for example [41]–[43] are needed to prevent reinforcing this geographic imbalance in successive generations.

## Reconciling Needs and Resolving Tensions

Resolving these tensions requires a collaborative, comprehensive, generation-spanning approach to global health education. A recent *Lancet* commission on professional medical education noted that education has stagnated in the face of growing and shifting health challenges; faculty are “essential to investing in future health dividends by training the next generation of health professionals” [11]. Indeed, faculty investment from all resource settings will be essential to lead rational and effective programs. Senior mentors from institutions in HI countries have expertise in the complex and high-tech care of diseases, advanced research methods, and innovative curriculum. However, these mentors do not provide the same breadth of experience as their developing country counterparts with respect to best practices in high disease burden, low-resource settings where these same technologies and medications are simply unavailable [44],[45]. Any global scale-up of education will require augmenting the bandwidth of leadership and experience of doctors trained in LMIC settings.

While academic mentorship and senior faculty are needed to lead this effort, investment must also overcome a “mid-level” leadership gap in LMIC academic centers. For both research and clinical medicine, this cadre of mid-level investigators and clinicians will create the visible and replicable pathway to international leadership for future students and junior trainees. The Network of African Health Science Academies states that a sustainable economic future for Africa lies in “strengthening the continent's scientific and technological capacity… [a goal that] can only be met if Africa educates and retains a critical mass of world-class scientists and technologists with the knowledge and expertise to address the continent's key scientific, technological and economic problems” [46]. A tenable path for career development will help buttress retention of indigenous physicians and researchers.

Considerable discussion has revolved around the importance of partnerships to integrate global health training [6],[47]–[49]. For example, the Swiss Commission for Research Partnerships (KFPE) published guidelines over a decade ago to guide best practices for how to establish mutually beneficial relationships [50]. However, these and other guidelines are not always heeded, and mobilizing complementary and equitable partnerships remains a challenge [51]. Whose interests are served through academic and other global health programs? The benefits for visiting residents and researchers are documented, including improved clinical skills, publications, and greater understanding of the challenges of delivering care in LMIC settings [21],[52]–[54]. There is less attention, however, devoted to the effects on recipient countries. Visiting trainees, for example, could potentially consume real financial and human resources without a clear benefit to host institutions [55]. Resources devoted to transportation, orientation, and acculturation need to be re-delivered to every incoming class of “rotators.”

Structured partnerships with devoted human resources and infrastructure foster integrative, supervised exchanges, which may help mitigate some of the intangible costs of volunteerism [47],[48]. The KFPE endorses the idea that not only the outcomes of research should be valued, but also the interaction between scientists and the public and how research impacts everyday life [50]. Along these lines, a number of such partnerships have developed between academic medical centers in HI countries and centers in LMIC settings. Cambridge University and its affiliated teaching hospital, Addenbrooke's, have partnered with Princess Marina Teaching Hospital in Gaborone, Botswana. Responding to the needs outlined by the Botswana hospital and Ministry of Health, the partnership has established common goals for education, research, and capacity building [56].

Medical institutions in HI settings, whose strengths are advanced practice standards, complex disease management, and scientific innovation, are natural allies to help buttress medical education and build capacity in partner countries. Drawing on their academic strengths, most HI countries' programs target support for three missions: health care delivery, research, and training their staff shoulder-to-shoulder with partner-country health providers. This “twinning” of professionals side-by-side encourages mobilization to fill human resource needs while simultaneously investing in capacity-building efforts and sustainable partnerships. To be effective in this mission, they rely on bi-directional teaching and training where developing local programs must be a priority [57]. For greater impact, programs need to be initiated and nurtured by both partner institutions rather than “inviting” in-country partners into plans that are already developed by the visiting partner. Success is measured two-fold: first by the quality of the experience for both the HI- and partner-country trainees, and second by the incremental improvement in in-country care, infrastructure, and/or research to which a trainee contributed.

While the detailed challenges of building effective research partnerships are beyond the scope of this discussion, they should not be minimized in the international setting. Important areas for mutual collaboration and outcomes include developing research priorities, technical capacity building, creating consensus across differing approaches to human subjects protection, establishing administrative and fiscal management structures, and maintaining reporting structures. Several organizations have dedicated themselves to just these challenges. Agencies such as the Wellcome Trust [41], WHO's Essence on Health Research [58], and the Council on Health Research for Development [59] are paying increasing attention to building institutional and overall system capacity for research. The focus on strengthening research capacity is originating from LMIC settings as well. The Initiatives for Strengthening Health Research Capacity in Africa is one such example [60].

## Investing in Health Leaders from All Educational Settings

Limited funding for education and capacity building remains a critical barrier to investing in leadership, defining long-term career trajectories, and strengthening partnerships. For example, a review of global health spending on development of health personnel, medical education, and training—which influence capacity building most directly—showed discouraging trends between 2000 and 2004; spending decreased from an already low 3% in 2000 to a mere 2% by 2004 [61]. Short-term rotations supported by discretionary funds or individual residency programs—as most are frequently established —are not pathways towards building sustainable global health leadership and a global health workforce.

New long-term funding structures are needed to prioritize capacity building and human resource development. The creation of national global health service corps is one option. In this model, economically and human resource–constrained countries could request faculty and skilled medical professionals to fill public sector health education vacancies for a sustained period of time. Donor nation government funding would be allocated in a public–private partnership to support these long-term placements [62],[63]. The National Health Service Corps is an existing model for service in rural and resource-constrained sites within the US [64]. For trainees from all settings, scholarships or loan forgiveness for service in public sector health system strengthening, training, and health care delivery would help provide essential support to young careers challenged by out-of-reach tuition or living costs [63]. Such programs could invest in the development of global health careers in both hemispheres and also accelerate scientific innovation towards a more meaningful, effective, and sustainable response to global health.

Because their investments are more flexible than those of public sector–funded programs, private philanthropic donations will also play an important role in supporting an effective global health response. Over the past decade, investments from private philanthropy through either individuals or foundations have grown exponentially [65]. This funding, however, is often disease or sector specific and is more focused on research and information gathering than on broad-based capacity building. With the increasing attention to health leadership needs, and specifically on faculty for teaching and training [11],[66], private funding opportunities must recognize in their funding priorities the need for health sciences leadership development as essential to long-term scientific advancement. Public-private-academic models between institutions in 1) the public sector of areas of high disease burden; 2) private philanthropy; and 3) HI academic centers can generate novel mechanisms to support innovation, clinical education, and technology transfer. The Baylor AIDS Global Health Service Corps in the US, funded by Bristol-Meyers Squibb, is such an example that has deployed physicians in public sector year-long placements for service delivery and training [67].

## Measuring Impact

Global health education and training programs must be evaluated on their progress towards reducing the global burden of disease. Programs need to develop mutually agreed upon criteria for balance of investments between partners in funding- and infrastructure-imbalanced partnerships. Programs should be evaluated on 1) leadership development, including the number of graduates from advanced degree programs and their retention in the field; 2) health care system strengthening, including clinical infrastructure, access to clinical services, workforce expansion, and improved health outcomes; and 3) scientific advancement, measured by new knowledge, research, treatments, technologies, or strategies to deliver care.

## Recommendations

Medical education training programs must engage the explosively growing interest in global health with a primary goal to reduce the global burden of disease through a sustainable investment in health systems and health care leadership. A collaborative, comprehensive approach to global health education with a generation-spanning timeline is needed. The core of any global health program requires balanced partnership, which leverages the strengths of both sides of the program toward pre-identified, mutually agreeable goals. Developing a new generation of leadership from both sides of the partnership through bi-directional training is paramount. Programs will need to redefine the expectations for training and include hosting partner country health professionals in HI countries for educational opportunities not readily available in LMIC countries. Programs must prioritize both developing senior leadership and supporting mid-level careers with a visible, replicable pathway for future students and junior trainees.

This paradigm for global health training will require collaboration amongst academic programs as well as government or private non-governmental agencies. As a field, we must not only create benchmarks of success, but we need to adopt them as well. A mutually acceptable accreditation process should be considered much as clinical specialties are subject to established standards. However, external support and new long-term funding structures that prioritize and monitor capacity building and human resource development are needed to realize these goals. As programs are evaluated, they should be measured on both their short-term success in scaling-up care and longer-term measures such as the number of graduates retained in sites, the research generated, or expansion of health system capacity.

## Conclusion

Success in reducing the global burden of disease will depend on how training programs manage the enthusiasm of trainees globally, and simultaneously create new incentives and training opportunities for health leadership in LMIC settings. Investments in scientific innovation to prevent and cure global diseases should be matched by those in the human resources required to discover and deliver innovations in prevention and treatment as well as train the next generation of leaders. This will require a long-term strategy that leverages strengths and talent from all settings. It will also require a generation spanning financial investment by HI countries and other multinational partners. The rising generation quickly needs a foothold on their potential, before their enthusiasm is extinguished by lack of direction, foresight, and opportunity.

## Abbreviations

HI: higher income
LMIC: low- and middle-income country
